# Suggestions to Patients

**Published:** 1892-09

**Authors:** 


SUGGESTIONS TO PATIENTS. By W. A. Yingling, M. D. Philadelphia: Bœricke & Tafel, 1892.

This small pamphlet is another one of the numerous magazines of directions for enabling patients to state their symptoms correctly. Two or three of these little books by different well-known homœopathic physicians have been before noticed in these pages. They are all useful in educating patients to accuracy in relating their symptoms. This is indeed a great task, as the people have been so long under an entirely different educational influence—that of the old school—that they do not understand the value of the minute differences of symptoms.

The author of the little book now under review is well known to our readers for his excellent articles that appear from time to time.

